# Evaluation of Three Commercial Interferon-γ Assays in a Bovine Tuberculosis Free Population

**DOI:** 10.3389/fvets.2021.682466

**Published:** 2021-06-10

**Authors:** Giovanni Ghielmetti, Patricia Landolt, Ute Friedel, Marina Morach, Sonja Hartnack, Roger Stephan, Sarah Schmitt

**Affiliations:** ^1^Section of Veterinary Bacteriology, Institute for Food Safety and Hygiene, University of Zurich, Zurich, Switzerland; ^2^Institute for Food Safety and Hygiene, University of Zurich, Zurich, Switzerland; ^3^Section of Epidemiology, University of Zurich, Zurich, Switzerland

**Keywords:** cattle, bovine tuberculosis, diagnosis, interferon-gamma assay, *Mycobacterium bovis*, *Mycobacterium avium* subsp *paratuberculosis*, *Mycobacterium avium* subsp *hominissuis*, *Mycobacterium persicum*

## Abstract

The interferon-γ assay has been used worldwide as an ancillary test for the diagnosis of bovine tuberculosis (bTB). This study aimed to describe, based on the bTB-free status in Switzerland, the difference of applying a more stringent cutoff point of 0.05 compared with 0.1 for bTB surveillance. Moreover, the effect of time between blood collection and stimulation, culture results, optical density values, and the influence of testing different breeds were evaluated. Blood samples from a total of 118 healthy cows older than 6 months were tested with three commercial interferon-gamma assays. To confirm the bTB-free status of the tested animals and to investigate potential cross-reactions with nontuberculous mycobacteria, pulmonary and abdominal lymph nodes in addition to ileal mucosa from each cattle were used for the detection of viable *Mycobacteria* spp. by specific culture. Significant differences regarding the proportion of false-positive results between the two Bovigam tests and between Bovigam 2G and ID Screen were found. Samples analyzed with Bovigam 2G were 2.5 [95% confidence interval (CI) 1.6–3.9] times more likely to yield a false-positive test result than samples analyzed with Bovigam TB. Similarly, the odds ratio (OR) for testing samples false-positive with ID Screen compared with Bovigam TB was 1.9 (95% CI 1.21–2.9). The OR for testing false-positive with ID Screen compared with Bovigam 2G was less to equally likely with an OR of 0.75 (95% CI 0.5–1.1). When using a cutoff of 0.05 instead of 0.1, the OR for a false-positive test result was 2.2 (95% CI 1.6–3.1). Samples tested after 6 h compared with a delayed stimulation time of 22–24 h were more likely to yield a false-positive test result with an OR of 3.9 (95% CI 2.7–5.6). In conclusion, applying a more stringent cutoff of 0.05 with the Bovigam 2G kit generates a questionable high number of false-positive results of one of three tested animals. Furthermore, specific breeds might show an increased risk to result false-positive in the Bovigam 2G and the ID Screen assays.

## Introduction

Bovine tuberculosis (bTB) is a chronic zoonotic disease caused by *Mycobacterium bovis* and *Mycobacterium caprae* (1). Cattle (*Bos Taurus*) are considered to be the main reservoir of bTB, and eradication programs worldwide focus primarily on this domestic species (2). Although such programs were able to significantly reduce the prevalence of the disease and some industrialized countries are considered to have official bTB-free status, specific geographical areas are still faced with this burden. Despite remarkable public and private efforts, the causes of this failure are multiple, including the varying clinical signs, intrinsic features of these bacteria such as the broad host spectrum and environmental resilience, the transmission modalities, and the absence of accurate *antemortem* diagnostic test applicable in the field (3–5).

The standard method for *antemortem* bTB detection and international trade of cattle is the tuberculin test, which involves the intradermal injection of bovine tuberculin purified protein derivative (PPDB) in the cervical area or the caudal fold of the tail (6). Skinfold thickness at the injection site is measured before and 72 h after PPDB is injected to calculate any increase. In addition, cutaneous reactions such as induration and swelling are evaluated. A more specific variant of the single intradermal test [single intradermal cervical comparative test (SICCT)] involves the additional injection of avian tuberculin (PPDA) into different sites, theoretically enabling distinction between animals infected with bTB and those responding to PPDA as a result of exposure to mycobacteria other than *Mycobacterium tuberculosis* complex (MTBC) (6). There are numerous known weaknesses associated with tuberculin skin testing, e.g., variations in specificity (Sp) and sensitivity (Se) related to different PPD products or even from batch-to-batch, the subjective injection and measurement ability of the test performer, and the necessity of restraining the animals twice (7, 8).

To overcome some of the mentioned drawbacks, a whole-blood cellular assay using a sandwich enzyme immunoassay (EIA) for the bovine cytokine interferon-gamma (IFN-γ) has been developed and used as a standalone or ancillary test to the intradermal tuberculin test for diagnostic purposes (9, 10). There are numerous advantages of the IFN-γ assay over the skin tests, including that the animals are captured only once, inconclusive tests can be readily repeated, quantification of the lymphocytes reaction to various stimulants is based on optical densities (ODs), and stimulation controls are included. Moreover, the IFN-γ assay may detect bTB-infected animals up to 60–120 days earlier than the single cervical tuberculin test (11–13).

The first IFN-γ assay (Bovigam TB, Thermo Fisher Scientific, Reinach, Switzerland) is now Office International des Epizooties-certified as an ancillary assay to the tuberculin test and may be authorized to maximize detection of infected cattle (Council Directive 64/432/ECC), including bTB-freedom certification for animals or products movement purposes and prevalence estimation (14). Switzerland was faced with bTB with two distinct outbreaks in 2013–2014 (15). At the time of writing, these events represent the last detection of *M. bovis* and *M. caprae* in domestic or wild animals in Switzerland. Although the first event was caused by the reemergence of an undetected *M. bovis* strain that circulated in the Swiss cattle population over at least 15 years, the second outbreak originated from cattle infected during summer pasturing in an Austrian endemic area (16, 17). Consequent test and cull measures based primarily on SICCT and comprehensive epidemiological contact-tracing investigation enabled the preservation of the official bTB-free status. Under these circumstances, the IFN-γ assay was used in the late stage of the epidemiological investigations as an ancillary test for non-negative SICCT animals.

In addition to the ongoing slaughterhouse surveillance through *postmortem* meat inspection, a nationwide monitoring program (LyMON) for early detection of bTB was started in 2013 (18). Meat inspectors were encouraged to submit altered bovine lymph nodes to the bTB reference laboratory for macroscopic inspection and culture for mycobacteria. Over an 8-year period, lymph nodes originated from 793 cows were sliced into thin sections (1–2 mm) and investigated for the presence of lesions compatible with bTB. Suspicious samples (*n* = 121) were homogenized and tested for the presence of MTBC DNA by real-time polymerase chain reaction (PCR) and cultured in mycobacterial selective media (15). Moreover, in 2014, a red deer monitoring program involving two bordering Swiss Cantons and the Principality of Liechtenstein was implemented (19). Over a 7-year period, retropharyngeal and mediastinal lymph nodes of 1,382 randomly and risk-based selected red deer, such as emaciated individuals or animals killed by motor vehicles, and 33 other wild animals (roe deer, fallow deer, fox, chamois, and Alpine ibex) were investigated macroscopically by trained personnel. Of these, a total of 412 wild animals were additionally tested for the presence of MTBC DNA by real-time PCR and cultured in mycobacterial selective media (15). No further bTB cases were detected after the two mentioned outbreaks in cattle, and although *M. caprae* is still endemic in the Austrian border region, none of the investigated red deer and other wild animals resulted positive.

Although the described surveillance measures testified that the Swiss population is currently free of bTB, punctual reintroductions through illegal imports or wildlife movements are possible. Therefore, in accordance with the European Food Safety Authority's scientific opinion, EIA cutoff thresholds should be evaluated in each country based on the local epidemiological conditions to ameliorate the accuracy of the IFN-γ assay (20). Hence, these modifications of the laboratory evaluation criteria may affect the Sp and Se of the assay. An estimated median Se of 87.6% (73–100%) and Sp of 96.6% (85–99.6%) were reported for the Bovigam TB kit based on 15 field studies conducted over a 15-year period (7). However, the median Sp value (96.6%) published by de la Rua-Domenech and colleagues (7) differs from the Sp values (6.9–74%) observed in further studies (21–23). These discrepancies may be the result of different testing conditions, laboratory procedures, and evaluation criteria and, most of all, may depend on the TB prevalence and the presence of nontuberculous mycobacteria (NTM), leading to cross-reactivity in the tested populations (24). Moreover, the IFN-γ production of specific breeds such as French bullfight cattle has been shown to be significantly lower than classical diary animals (4, 24).

Recently, a new IFN-γ assay has become commercially available (ID Screen Bovine Tuberculosis IFN-γ, IDvet, Grabels, France), and it has already been extensively used in different countries including Switzerland. However, independent evaluations on the test accuracy under field conditions are either not publicly available or scarce, showing low Se (36.7%) when applying the cutoff recommended by the manufacturer or slightly better values (49.0–56.0%) using more stringent cutoff thresholds (25).

In their scientific opinion, the expert panel convened on the use of the IFN-γ test for the diagnosis of bTB highlighted the necessity to harmonize the assay protocol (20). In this regard, various critical points need to be evaluated, such as the time between blood collection and stimulation, antigens used and their concentrations, interpretation criteria including cutoff values, and finally, the inclusion of stimulation control and EIA control. Nevertheless, specific epidemiological conditions, such as exposure to environmental mycobacteria in certain geographical areas, possibly negatively influence the test accuracy (26–28).

Besides the members of the MTBC, over 190 species of NTM have been described (www.bacterio.net/mycobacterium.html). Several NTMs are commonly encountered in the environment and have been isolated from a variety of sources, such as water, feed, soil, dust, aerosol, protozoa, and animals, including cattle (29, 30). Of these, more than 60 species are known to be opportunistic pathogenic to humans and other mammals, and human infections with these emerging pathogens are now more common than tuberculosis in industrialized countries (31–33).

To avoid cross-reactive immune responses in cattle exposed to NTM, PPDA is injected in the SICCT or included in first-generation IFN-γ assays. However, in geographical areas where the dominant NTM shares epitopes with MTBC members, more specific diagnostic markers have been identified (34–36) and implemented in second-generation IFN-γ assays and subsequent versions (37, 38). Among these, antigen cocktails containing the 6-kDa early secretory antigenic target- and 10-kDa culture filtrate protein-derived peptides have gained great importance for their presumptive MTBC specificity (39). Despite earlier evidence to the contrary, orthologs of 6-kDa early secretory antigenic target and 10-kDa culture filtrate protein have now been shown to be present in frequently isolated NTM, such *Mycobacterium smegmatis, Mycobacterium kansasii, Mycobacterium persicum, Mycobacterium marinum, Mycobacterium szulgai, Mycobacterium gastri*, and *Mycobacterium flavescens* (34, 40–43). The specificity of the available commercial IFN-γ assays is therefore questionable in geographical areas where cattle may be exposed to NTM, influencing the test accuracy. An increased number of false-positive (FP) tested animals can result in reduced stakeholders' acceptance of control measurements, undermining the credibility of the involved authorities.

This study aimed to describe, based on the epidemiological situation (bTB-free status) in Switzerland, the difference of applying a cutoff point of 0.05 compared with 0.1 and considering the ethical implications, i.e., if a more stringent cutoff is ethically acceptable for bTB surveillance and import/export of livestock. Additionally, the effect of time between blood collection and stimulation, culture results, and OD values were evaluated.

## Materials and Methods

### Selection Criteria and Collection of Blood Samples

From a total of 118 randomly selected healthy cows older than 6 months originating from 101 different premises and 14 Swiss Cantons, two blood samples were collected *antemortem*. No more than two cows originating from the same premise were included in the study. Three different breeds covered ~90% of the animals, with Holstein/Red Holstein (*n* = 36) being the most represented group, followed by Swiss Fleckvieh/Simmental (*n* = 35) and Swiss Brown (*n* = 32). The remaining animals were Montbéliard (*n* = 8) Normande (*n* = 2), one Charolais, one Limousin, and three mixed breeds. Of these, 90% were regularly moved to pastures in mountain regions during the summer months. Before sampling, animals were stabled in the proximity of the abattoir during the morning of the day before being killed, and heparinized blood was collected by trained personnel from the caudal vein ≥3 h after delivering. Each sample was immediately transported unchilled to the laboratory.

### Culture and Identification of Mycobacteria

To confirm the bTB-free status of the tested 118 animals and to detect potential cross-reactions with NTM species, a pool of pulmonary lymph nodes (left bronchial and caudal mediastinal) and a pool of intestinal tissues (ileal mucosa and jejunal/cecal lymph nodes) from each cattle were collected immediately after killing. The pulmonary lymph nodes were cultured at 37°C for 8 weeks as described elsewhere (30). The pool of intestinal tissues was cultured at 37°C for 16 weeks on Herrold's Egg Yolk Agar with mycobactin J and ANV (BD, Basel, Switzerland), on BBL Stonebrink agar slants (BD) and on liquid MGIT supplemented with PANTA (BD) and mycobactin J (IDvet) as culture media.

All tubes showing growth of presumptive mycobacterial colonies were investigated for acid-fast bacilli after Ziehl–Neelsen staining. The colonies were identified using matrix-assisted laser desorption/ionization-time of flight mass spectrometry, and presumptive positive *Mycobacterium avium* subsp. *paratuberculosis* (MAH) colonies were confirmed by the ID Gene Paratuberculosis Duplex PCR (IDvet). For three isolates, sequencing of two housekeeping genes (*hsp65* and 16S rRNA) was performed (30). DNA sequencing was performed at Microsynth (Balgach, Switzerland). The resulting sequences were assembled using CLC Genomics Workbench 7.5.1 (Qiagen), and BLAST similarity searching for multiple sequence alignment was performed (https://blast.ncbi.nlm.nih.gov/Blast.cgi).

### Interferon-γ Assays

The commercial IFN-γ kits Bovigam TB (Thermo Fisher Scientific), Bovigam 2G (Thermo Fisher Scientific), and ID Screen Bovine Tuberculosis IFN-γ (ID Screen) were tested for each blood sample according to the manufacturers' instructions. The blood samples were divided into four aliquots and incubated with PPDB, PPDA, both in addition to pokeweed mitogen (PWM) (Prionics, Lelystad, The Netherlands for Thermo Fisher Scientific; CZ Veterinaria, Porriño, Spain for IDvet) and phosphate-buffered saline (NIL) as positive and negative stimulation controls, respectively. Two different stimulations times for each blood sample were performed, and these occurred within 6 and 22–24 h after collection, respectively. Released IFN-γ was measured using the enzyme-linked immunosorbent assay kit provided by the respective manufacturer.

Interpretation of the three kits was performed using the following criteria, with the mentioned ODs representing mean values:

- Criterion 1: positive test outcome = OD_PPDB_ – OD_NIL_ ≥ 0.05 and OD _PPDB_ – OD _PPDA_ ≥ 0.05
- Criterion 2: positive test outcome = OD_PPDB_ – OD_NIL_ ≥ 0.1 and OD_PPDB_ – OD_PPDA_ ≥ 0.1

The results of the ID Screen kit were additionally evaluated using the S/P ratio, as recommended by the manufacturer.

- Positive test outcome: $\frac{S}{P}\;=\;\left(\frac{\text{O}{\text{D}}_{\text{PPDB}}-\text{O}{\text{D}}_{\text{PPDA}}}{\text{O}{\text{D}}_{\text{ELISA positive control}}-\text{O}{\text{D}}_{\text{ELISA negative control}}}\right)*100\geq \;35\text{\%}.$

To assess the viability of T cells, stimulation with PWM was included for each test, and OD values of samples that not fulfilled the following criteria were interpreted as invalid. For the ID Screen kit, the following interpretation was adopted: $\frac{\text{O}{\text{D}}_{\text{PWM}}}{\text{O}{\text{D}}_{\text{ELISA positive control}}\;-\text{ O}{\text{D}}_{\text{ELISA negative control}}}\geq 0.5,$ whereas for both Bovigam variants, mean values of OD_PWM_ – OD_NIL_ were supposed to be ≥0.5.

Moreover, animals responding to unspecific stimulation where excluded if their mean OD_NIL_ was ≥0.3.

### Statistical Methods

With the aim to assess if the proportion of FP test results (i) differed between the three IFN-γ assays, (ii) was affected by the time elapsed between sampling and stimulation (6 and 22–24 h), (iii) and accounting for two different cutoffs (0.05 and 0.1), a model was fit with generalized estimating equations with the function geeglm from the package geepack in R (44). To account for potential within-animal clustering, an exchangeable correlation was chosen in the marginal model. Adjustment for multiple comparisons between the three IFN-γ assays was performed with Tukey's approach from the multcomp package (45). The resulting effect sizes are presented in the form of odds ratios (ORs) with their corresponding 95% confidence intervals (CIs) and *p*-values, adjusted for multiple comparisons.

Based on culture outcome, three different groups were defined as follows: MAP (*n* = 6), MAH (*n* = 14), and culture-negative animals (*n* = 94). Responses to PPDA (OD_PPDA_) of the three groups were investigated for the different kits and stimulation within 6 h using a linear mixed-effects model with animals as a random effect (nlme-package) (46). The differences of the mean ODs PPDA-NIL of the three groups were compared for each kit. In addition, the effect of time between blood collection and stimulation (6 and 22–24 h) on the mean OD_PWM_ values was assessed for the three kits using the same model as for PPDA. Fisher's exact test using GraphPad Prism 9.1.0 (GraphPad Software) was performed to evaluate a possible association between positive test outcome and animal breed for the three major breed groups after 6 h stimulation.

## Results

### Culture and Identification of Mycobacteria

All the tissue samples were negative for MTBC, whereas NTM was found in 17 of the 118 cultured pulmonary lymph node pools with MAH as the predominant species (*n* = 14). In the remaining three pools, *M. persicum, Mycobacterium lentiflavum*, and a member of the *Mycobacterium chimaera/intracellulare* group were isolated. In six of the 118 intestine tissue pools, MAP was cultured, and one animal was positive for *Mycobacterium engbaekii*.

### Interferon-γ Assays

**Figure 1:**
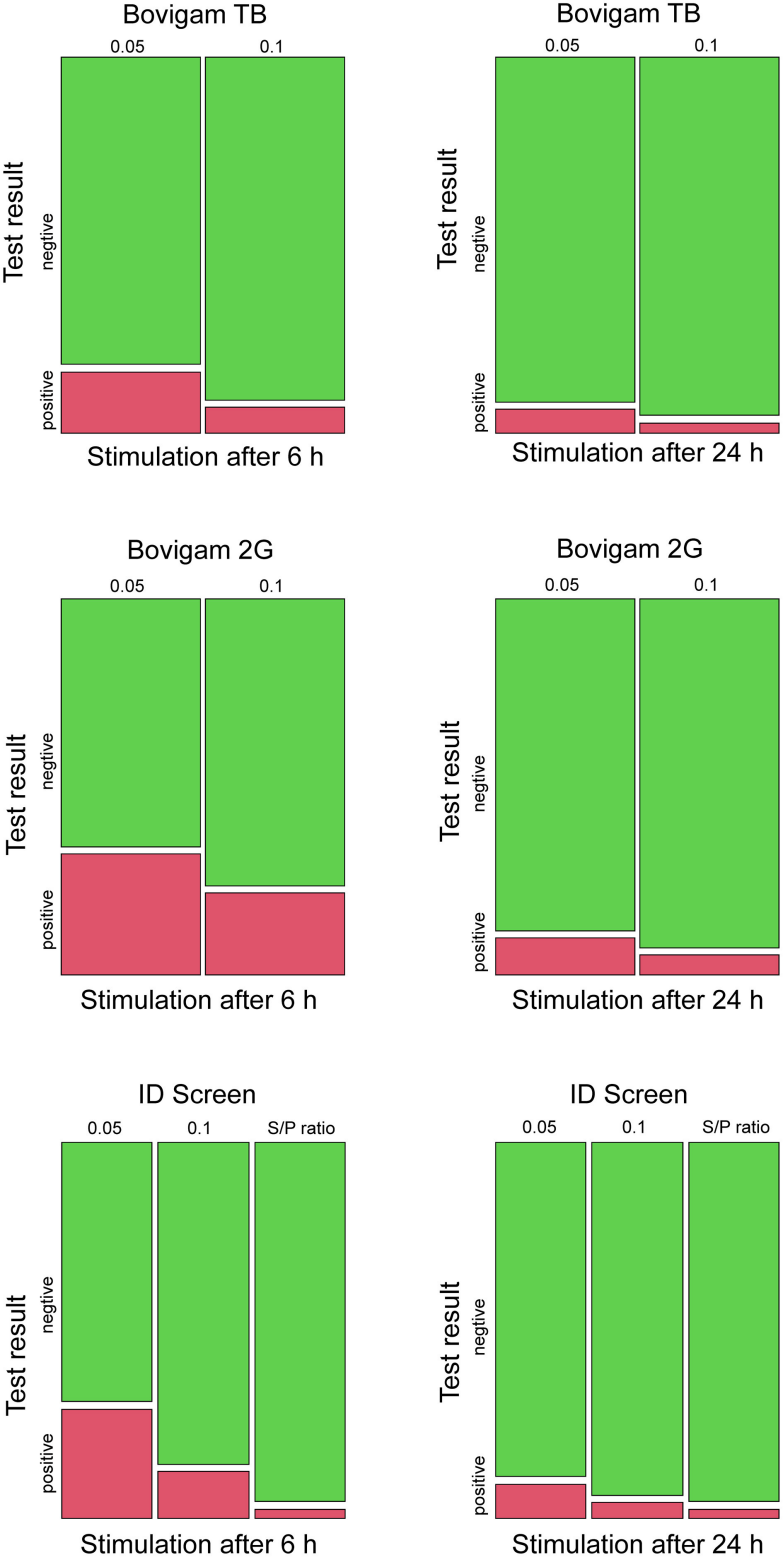
Mosaic plots for the test outcomes using three different kits (Bovigam TB, Bovigam 2G, ID Screen), stimulation times (6 and 24 h), and cutoffs (0.05, 0.1, S/P ratio). Results are displayed in green and red for negative and positive responses, respectively.

The test outcomes for each kit, stimulation time, and cutoffs are resumed in Figure 1. The animal positive for *M. persicum* showed a clear positive result with all three kits, stimulation times, and cutoffs. Cattle tested positive for MAP were negative with all kits and test conditions except for two FP results with the lower cutoff of 0.05 (all three kits). The majority of the MAH-positive animals showed negative results with the IFN-γ assay; only two animals were positive using the stricter cutoff of 0.05 (Bovigam 2G and ID Screen), whereas one animal was also positive using the cutoff of 0.1 and the Bovigam 2G kit.

Taken the culture as a gold standard and assuming the likelihood of an MTBC infection in Swiss cattle as very unlikely, every positive test result was assumed to be an FP result. The false-positive rate (FPR) for the three kits, stimulation times, and cutoff were evaluated. The ID Screen kit with the S/P ratio evaluation showed the best performance with an FPR of 2.5% (95% CI 0.8–7.2), independently from the stimulation times. The other two kits showed high amounts of FP results, especially when applying the more stringent cutoff (criterion 1) and the stimulation time of 6 h after blood collection, e.g., FPR 32.7% (95% CI 24.2–41.3) for the Bovigam 2G (Table 1).

**Table 1:** Specificity of the three commercial kits assessed with two different times after stimulation and OD cutoffs. Specificity (95% CI), time after stimulation shown in hours after blood collection, and OD cutoffs including criterion 1 (0.05), criterion 2 (0.1), and S/P ratio for the ID Screen kit were evaluated.

**Kit**	**Stimulation time**	**Criterion 1**	**Criterion 2**	**S/P ratio**
Bovigam TB	6 h	83.33 (75.20–89.66) [4]^†)^	92.98 (86.64–96.92) [4]	/
	22–24 h	93.52 (87.10–97.35) [10]	97.22 (92.10–99.42) [10]	/
Bovigam 2G	6 h	67.26 (57.79–75.79) [5]	77.88 (69.10–85.14) [5]	/
	22–24 h	90.09 (82.96–94.95) [7]	94.59 (88.61–97.99) [7]	/
ID screen	6 h	70.34 (61.23–78.39) [1]	87.29 (79.90–92.71) [1]	97.46 (92.75–99.47) [1]
	22-24 h	90.68 (83.93–95.25) [0]	95.76 (90.39–98.61) [0]	97.46 (92.75–99.47) [0]

† *)Number of invalid tests results excluded from the analysis according to the interpretation criteria described in the Materials and Methods section are displayed in square brackets*.

### Comparison of Kits, Cutoffs, and Stimulation Time

The applied statistical models enabled a comparison of the three kits, taking into account the two different cutoffs and stimulation times. Based on the results of the generalized equation estimates, significant differences regarding the proportion of FP between the two Bovigam tests and between Bovigam 2G and ID Screen were found. Samples analyzed with Bovigam 2G were 2.5 (95% CI 1.6–3.9) times more likely to yield an FP test result than samples analyzed with Bovigam TB. Similarly, the OR for testing samples FP with ID Screen compared with Bovigam TB was 1.9 (95% CI 1.21–2.9). The OR for testing with ID Screen compared with Bovigam 2G was 0.75 (95% CI 0.5–1.1). When using a cutoff of 0.05 instead of 0.1, the OR for an FP test result was 2.2 (95% 1.6–3.1). For samples with a stimulation time of 6 h compared with 22–24 h, the OR of testing FP was 3.9 (95% CI 2.7–5.6).

### Culture Outcome and OD_PPDA_ Values

The OD values in response to PPDA of the MAP-(*n* = 6) and MAH-(*n* = 14) infected animals were compared with the corresponding values of the culture-negative animals. No significant variation could be observed between the three groups (*p* = 0.166). Because the number of animals with MAP is low, this result should be considered with caution due to power.

### Stimulation Time and OD_PWM_ Values

Delay in blood stimulation (22–24 h) caused a significant reduction of the mean OD_PWM_ obtained. This phenomenon was particularly evident for the ID Screen kit (overall mean OD_PWM6h_ 2.94 and OD_PWM22−24*h*_ 2.36). The smallest effect of time stimulation was observed for the Bovigam TB kit (overall mean OD_PWM6h_ 1.40 and OD_PWM22−24*h*_ 1.29); this kit had the lowest mean OD values and also the most invalid results (*n* = 10; 8.5%) compared with the other two kits (Table 1). The Bovigam 2G kit had two invalid results because of low mean OD_PWM22−24*h*_ values (overall mean OD_PWM6h_ 2.58 and OD_PWM22−24*h*_ 2.17), by contrast, five invalid result for this kit were observed due to high mean OD_NIL6h/22−24*h*_ values (≥0.3). Using the described interpretation criteria, the ID Screen kit showed one invalid result due to a high mean OD_NIL6h_ value (≥0.3) in one animal.

### Association Between Positive Test Outcome and Animal Breed Groups

**Figure 2:**
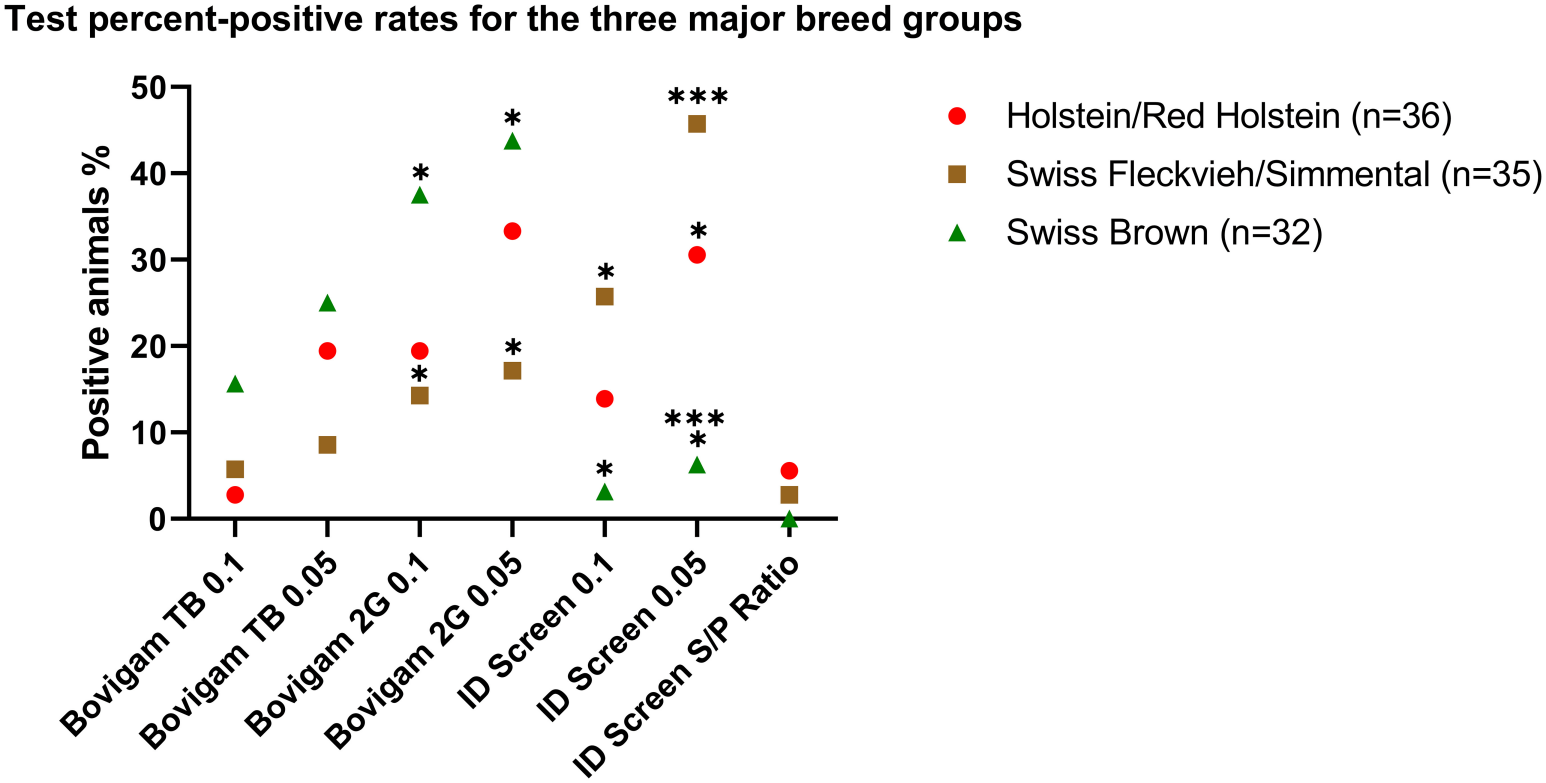
Test percent-positive rates for the three major breed groups. Data are shown as a positive percentage of each breed group for the tested kits at 6 h stimulation. Fisher's exact test was used to evaluate the different groups. *P < 0.05; ***P < 0.001.

Three major animal breed groups were investigated, with Holstein/Red Holstein (*n* = 36) being the most represented group, followed by Swiss Fleckvieh/Simmental (*n* = 35) and Swiss Brown (*n* = 32). As shown in Figure 2, by using a 0.1 cutoff and the Bovigam 2G kit, animals classified as Swiss Brown were more likely to test positive compared with Swiss Fleckvieh/Simmental (37.50 and 14.29%, respectively, *P* < 0.05). This difference was even more evident by applying the more stringent 0.05 cutoff, with 43.75 and 17.14% positive Swiss Brown and Swiss Fleckvieh/Simmental, respectively (*P* < 0.05). The opposite effect was observed by using the ID Screen kit (0.1 cutoff), with 25.71% of the Swiss Fleckvieh/Simmental animals resulting positive compared with 3.13% of the Swiss Brown. This phenomenon was exacerbated by applying the more stringent 0.05 cutoff, with 45.71 and 6.25% positive Swiss Fleckvieh/Simmental and Swiss Brown, respectively (*P* < 0.001). No statistically significant difference was observed between the test outcome and the three breed groups for the Bovigam TB kit.

## Discussion

According to the Swiss technical instruction for bTB, the IFN-γ assay is applied in specific epidemiologically relevant situations where the intradermal tuberculin skin test leads to inconclusive results. Cattle are the major livestock species in Switzerland, with roughly 1.5 million animals (Federal Statistical Office, census 2019). Approximately two-thirds of the Swiss cattle industry is dedicated to dairy production, and the average lifespan of a dairy cow in Switzerland is 6.2 years, giving birth on average 3.7 calves in a lifetime (47).

The agents causing bTB have been isolated from numerous different domestic and wild animal species, with the latter possibly covering long distances and spreading the disease (48, 49). This results in continuous interspecies transmissions from wild animals to livestock and *vice versa*, hindering national and international eradication programs (16, 50–52). Badger (*Meles meles*), free-ranging red deer (*Cervus elaphus*), and wild boar (*Sus scrofa*) are the most relevant known wild animals acting as a reservoir of bTB in Europe.

Accuracy of the IFN-γ assay for diagnosis of bTB varies considerably according to the literature and depends on the epidemiological settings of the tested population, the laboratory procedure, and the evaluation criteria adopted. The use of more stringent cutoff thresholds can increase the Se of the assay but may negatively influence the Sp. To date, different evaluation criteria, including thresholds, are currently used by European Member States (20). In our hands, statistically significant differences were observed for the different kits concerning the test outcome (Bovigam TB *vs*. Bovigam 2G and ID Screen *vs*. Bovigam TB), whereas the mean OD values in response to PPDA of the three culture groups (MAP, MAH, and culture-negative animals) showed no statistically significant differences.

Delay in the stimulation of the blood samples >6–8 h has been described to negatively affect IFN-γ assay performance, significantly decreasing ODs of tuberculin skin-test reactor and non-reactor animals (9, 53). The mentioned delay was deliberately included in the project to simulate a sample delivery overnight in comparison with immediate blood stimulation. Statistically significant reduction of the OD values obtained from samples PWM-stimulated within 6 h and after 22–24 h was observed for all three kits tested in the present study. This is in line with previous observations in cattle and goats (53, 54) and could negatively affect the Se of the assay due to the reduction of viable T cells in the sample.

As previously shown for *M. kansasii* (55), exposure to *M. persicum* may lead to FP results, independently from the assay used, whereas, based on the present findings, MAH and MAP seem to play a minor role in the cross-reaction. Hence, contrary to our expectations, no significant effect on the PPDA median OD values was observed between the three groups (MAP, MAH, and culture-negative) analyzed. This may be due to previous contacts of the negative culture group with mycobacteria sharing common antigens with those included in the PPDA cocktail. Among these, MAH, a ubiquitous environmental saprophyte frequently isolated from water, soil, and various animal species, including cattle, is to be mentioned and possibly plays a crucial role (30, 56).

Similar to *M. kansasii, M. persicum* shows marked homologies in surface protein expression, e.g., CFP-10 and ESAT-6, to MTBC members (42). Based on these findings, the inclusion of more specific antigens as proposed by second-generation assays may not overcome cross-reactivity issues due to NTM.

Alarming high amounts of FP results were observed using the two Bovigam assays, especially when applying the more stringent cutoff (criterion 1) and the stimulation time of 6 h after collection. For instance, using the Bovigam 2G kit, an FPR of 32.7% (95% CI 24.2–41.3) was determined, meaning that one-third of the tested animals are supposed to be classified as positive, although MTBC was not cultured in the tested population.

According to the manufacturer's recommendations, 8.5% (*n* = 10) and 5.9% (*n* = 7) of the cattle showed an invalid result when blood samples were stimulated 22–24 h after collection and tested with the Bovigam TB and the Bovigam 2G, respectively. For the ID Screen kit, one invalid result was observed. Under realistic conditions, an invalid result would require an additional farm visit for blood sample collection, resulting in increased time and cost efforts. Previous reports showed that the collection of blood samples before stunning or even at the commencement of exsanguination is a reliable method for accessing bTB infection using the IFN-γ assay (57). A negative effect on the IFN production due to holding and handling procedures in the present study, however, cannot be excluded (24). Thus, the number of invalid results should be interpreted with caution.

Genetic influence of the breed and the outcome of the SICCT test have been reported (58). A similar impact on the Bovigam TB test result, however, has only been demonstrated in particular cases (4). Although the present findings need further confirmation with larger animal numbers, specific breeds might show an increased risk to result in FP in the Bovigam 2G and the ID Screen assays, whereas, in accordance with previous observations (59), this was not seen for the Bovigam TB kit.

Epidemiological aspects, such as farming conditions, have been shown to play a pivotal role in the *antemortem* bTB diagnostic (4, 24). Swiss cattle spend the summer months grazing on Alpine and pre-Alpine pastures, possibly resulting in prolonged close contact with environmental NTM. Considering the particular epidemiological context of the present study, similar low individual Sp values of the IFN-γ assay were observed in neighboring countries such as France and Germany (22, 23). This suggests that the observed local cross-reactivity of NTM and possibly the negative effect on the assay due to specific breeds may not be restricted to Switzerland.

In conclusion, the general application of the IFN-γ assay and, in particular, the use of more stringent interpretation criteria should be carefully evaluated. Under specific settings such as testing animals originating from herds with bTB-positive cases or import from endemic areas, the assay ensures the detection of additional infected animals and, in some cases, permits their earlier recognition compared with the tuberculin test (12). In the authors' opinion, despite the unsatisfactory Sp observed in the present study, interpretation criteria should be adapted depending on the epidemiological context. For bTB-epidemiologically linked herds, Se should be prioritized over Sp, applying the more stringent cutoff. Conversely, for surveillance purposes within a cattle population with low bTB prevalence, however, Sp should be prioritized using a more suitable cutoff or an alternative test. Within the context of a notifiable and zoonotic disease such as bTB, however, culling of infected animals to stop the spread of the causative pathogen and to reduce animal suffering is considered necessary for disease control. Preemptive culling and culling due to compromised welfare due to transport restrictions raised ethical concerns. These concerns are resumed in the EU directive 2003/85/EC: “One of the Community's tasks in the veterinary field is to improve the state of health of livestock, thereby increasing the profitability of livestock farming and facilitating trade in animals and animal products. At the same time the Community is also a Community of values, and its policies to combat animal diseases must not be based purely on commercial interests but must also take genuine account of ethical principles” (46). Still, it is not clear what according to the EU legislation is meant by “taking ethical principles into genuine account.” One possible approach to gain clarity is to consider three standard ethical principles, which are respect for well-being, autonomy, and fairness or justice (47). Thus, the ethical question here is to assess if applying a more stringent cutoff for IFN-γ tests negatively affects the well-being, autonomy, and fairness of cows, farmers, and other potential stakeholders. Although the arbitrary value of the culled animals is currently reimbursed by the Swiss Government, the individual value of single animals for their farmers is often higher and results from decades of meticulous genetic selection. Briefly sketched, when applying a more stringent cutoff threshold, the well-being of the cows and the farmers will be affected because—based on the present OR of 2.2 (95% CI 1.6–3.1)—the odds or chance of an FP test result is two times more likely when reducing the cutoff from 0.1 to 0.05. A harmonized or generally prescribed cutoff of 0.05—irrespective of the local epidemiological situation, which is determined by the local prevalence of bovine TB, the occurrence of other cross-reacting NTM, and the tested cattle breed—might affect the principle of autonomy, presumably mostly relevant for the local veterinary authorities. As cows living in geographical areas with a higher occurrence of certain NTM species, they might be more likely to obtain an FP test result, which would affect the principle of fairness and justice. Briefly summarized, following the principles approach, the general application of a stringent cutoff of 0.05 compared with 0.1 is ethically questionable. Still, based on the results of our study, with a cutoff of 0.1 and the stimulation time of 6 h, the point estimates of the specificities of ID Screen and Bovigam 2G are below 90%. For the purpose of illustration, in an epidemiological setting with a true TB prevalence of 10%, assumed test sensitivity and specificity of 95 and 90%, the probability that a cow with a positive test result is truly infected is 51%. If the true prevalence is 5%, this probability is reduced to 33.3%.

Modifying the cutoff with the aim to increase the specificity is also not a feasible solution, as this would potentially lead to a decrease in sensitivity. A potential TB outbreak might be detected later, affecting subsequently more cows, causing more welfare losses in both cows and farmers. The application of a more stringent cutoff during the clarification of an outbreak may result in a significantly high number of animals with negative *postmortem* tests, such as RT-PCR or culture, greatly diminishing the reliability of positive IFN-γ results and consequently the long-term compliance of farmers. Based on our results regarding the proportion of FP and the presence of NTM potentially causing FP test results, we suggest evaluating the IFN-γ assay in light of the local epidemiological situation (20). Thus, a better understanding of the local epidemiological situation, including the identification of breeds that are more likely to react FP with specific test assay, is crucial. Monitoring programs such as LyMON provide essential data on the possible reoccurrence of bTB, and the present findings highlight an alarmingly high number of FP reactors. Moreover, transparency of data generated from surveillance studies is essential for the establishment of an international standard based on OD-values.

Within the context of One Health, the well-being, autonomy, and fairness of the involved parties such as dairy farmers, consumers, and cows should be considered. Among others, single aspects including food safety, animal welfare, and the intrinsic value of animals are some of the interests to be evaluated before more stringent diagnostic tests are officially approved.

## Conclusion

The application of a more stringent threshold leads to a questionable high number of FP results. Depending on the epidemiological context, including cross-reactive NTM in specific geographic areas, expected bTB prevalence, consequences of a positive test, the tested cattle breed, and the assay threshold should be carefully selected. If necessary, the inclusion of more specific antigens is to be considered.

## Data Availability Statement

The raw data supporting the conclusions of this article will be made available by the authors, without undue reservation.

## Ethics Statement

All animals included in this study were sampled in accordance with the Swiss Act SR 455. The animal testing was approved by the Animal Welfare Committee of the Canton of Zurich under license number ZH185/18.

## Author Contributions

GG, SS, and RS designed and coordinated the study. PL, MM, GG, and UF performed the experiments. SH and GG conceived and carried out the statistical analyses. GG, SS, and SH drafted the manuscript. All authors read and approved the final manuscript.

## Conflict of Interest

The authors declare that the research was conducted in the absence of any commercial or financial relationships that could be construed as a potential conflict of interest.
